# Association of fruits, vegetables, and fiber intake with COVID-19 severity and symptoms in hospitalized patients: A cross-sectional study

**DOI:** 10.3389/fnut.2022.934568

**Published:** 2022-09-29

**Authors:** Kiana Tadbir Vajargah, Nikan Zargarzadeh, Armin Ebrahimzadeh, Seyed Mohammad Mousavi, Parnia Mobasheran, Pari Mokhtari, Habib Rahban, Mihnea-Alexandru Găman, Camellia Akhgarjand, Mohsen Taghizadeh, Alireza Milajerdi

**Affiliations:** ^1^School of Medicine, Tehran University of Medical Sciences, Tehran, Iran; ^2^Research Center for Biochemistry and Nutrition in Metabolic Diseases, Institute for Basic Sciences, Kashan University of Medical Sciences, Kashan, Iran; ^3^Department of Community Nutrition, School of Nutritional Sciences and Dietetics, Tehran University of Medical Sciences (TUMS), Tehran, Iran; ^4^Department of Pharmacology and Experimental Therapeutics, Louisiana State University Health Sciences Center, New Orleans, LA, United States; ^5^Department of Pediatrics, The Saban Research Institute, Children's Hospital Los Angeles, Los Angeles, CA, United States; ^6^Cardiovascular Research Foundation of Southern California, Beverly Hills, CA, United States; ^7^Southern California Medical Education Consortium, Temecula Valley Hospital, Universal Health System, Temecula, CA, United States; ^8^Faculty of Medicine, “Carol Davila” University of Medicine and Pharmacy, Bucharest, Romania; ^9^Center of Hematology and Bone Marrow Transplantation, Fundeni Clinical Institute, Bucharest, Romania

**Keywords:** fruits, vegetables, dietary fiber, COVID-19, severe disease, infectious disease

## Abstract

**Background and aims:**

Fruits and vegetables are rich in fiber and a good source of anti-inflammatory and immune-boosting vitamins, minerals, and antioxidants. We investigated the association between fruits, vegetables, and fiber intake and severity of COVID-19 and related symptoms in hospitalized patients.

**Methods:**

A total of 250 COVID-19 hospitalized patients aged 18 to 65 years were recruited for this cross-sectional study in Kashan, Iran, between June and September of 2021. Dietary intakes were assessed using an online validated 168-item food frequency questionnaire (FFQ). COVID-19 severity and symptoms were evaluated using the National Institutes of Health (NIH) COVID-19 Treatment Guidelines. Moreover, we examined COVID-19 symptoms, inflammatory biomarkers, and additional factors.

**Results:**

The mean age of participants was 44.2 ± 12.1 years, and 46% had severe COVID-19. Patients with higher consumption of fruits (OR: 0.28; 95% CI: 0.14-0.58, *P*-trend <0.001), vegetables (OR: 0.33; 95% CI: 0.16-0.69, *P*-trend <0.001), and dietary fiber (OR: 0.25; 95% CI: 0.12-0.53, *P*-trend <0.001) had lower odds of having severe COVID-19. In addition, they had shorter hospitalization and convalescence periods, lower serum C-reactive protein (CRP), and a reduced risk of developing COVID-19 symptoms such as sore throat, nausea and vomiting, dyspnea, myalgia, cough, weakness, fever, and chills.

**Conclusion:**

Higher consumption of fruits, vegetables, and fiber was inversely linked with COVID-19 severity, clinical symptoms, hospitalization and convalescence duration, and CRP concentrations. The results should be interpreted with caution in light of the limitations, and prospective cohort studies are required to further evaluate these findings.

## Introduction

The coronavirus disease 2019 (COVID-19), which was caused by the novel coronavirus SARS-CoV-2, had a major impact on the lives of people around the world (1). Since its discovery in late 2019, it has resulted in significant mortality and morbidity worldwide (2). By 28 December 2021, the WHO had reported more than 279 million cases and 5.3 million deaths (3). Over 6.1 million confirmed COVID-19 cases and 131,400 COVID-19 deaths were reported in Iran as well (4). COVID-19 infection manifests from clinically asymptomatic to critical and life-threatening illness (5). COVID-19 also affects all organ systems in the human body, including the hematological system (alterations of the hemostasis), the pulmonary system (acute hypoxic respiratory failure, pneumonia, pulmonary embolism, and pulmonary fibrosis), the cardiovascular system (coronary artery atherosclerosis and myocardial infarction), the nervous system (stroke, impaired consciousness and encephalopathy, convulsions, visual impairment, and ataxia), and the gastrointestinal system (6). In addition, SARS-CoV-2 infection placed a great financial burden to the global economy and healthcare systems (7–9).

Diet and other lifestyle factors are well-known risk factors for obesity (10), hypertension (11, 12), and diabetes (13–15), all of which have been identified as potential risk factors for severe COVID-19 (16, 17). Adherence to low in fiber, fruits, and vegetables dietary patterns has been associated with an increased risk of these chronic diseases (18–21). In contrast, adherence to a healthy diet rich in fruits and vegetables is essential for proper immune function (22, 23). In a systematic review and meta-analysis of 83 studies, Hosseini et al. showed that high fruits and vegetables consumption is associated with lower levels of pro-inflammatory mediators and enhanced immune responses (24).

The Middle Eastern diet is characterized by a greater intake of carbohydrate (mostly derived from refined grains), trans and saturated fatty acids, a lower intake of fruits and vegetables, and absence of alcohol intake (25). To the best of our knowledge, no study has been conducted to examine the association between dietary fiber, vegetables, and fruit intake with the risk of severe COVID-19 and its symptoms. Therefore, we aimed to investigate the potential association between dietary fiber intake, fruit, and vegetables and the odds of severe COVID-19 and its symptoms in a cross-sectional study.

## Materials and methods

### Participants

This cross-sectional study included 250 COVID-19 patients aged 18 to 65 years who had recovered from the disease. Simple random sampling was used to select the study population from the Shahid Beheshti Hospital in Kashan, Iran, between June and September 2021. The study protocol was approved by the ethics committee of Kashan University of Medical Sciences (registration no. IR.KAUMS.MEDNT.REC.1400.048). The study was discussed with all participants and written consent was obtained from them.

Initially, the medical records of 600 patients with COVID-19 were reviewed, with consideration given to patients diagnosed within the past three months. In total, 350 of them were excluded due to the following exclusion criteria. Patients with diseases other than COVID-19, medical history of chronic diseases such as diabetes mellitus and cardiovascular disease, as well as conditions with potential impact on COVID-19 severity, body mass index (BMI) > 40 kg/m^2^, pregnant or breastfeeding women, active smokers, those were taking dietary supplements more than twice a week before the initial diagnosis of COVID-19; followed specific diets; taking medications with potential effects on the respiratory system, e.g., fluticasone and flunisolide, and those with missing information in their medical records were not included. Consequently, 250 patients were analyzed and included in this study.

### Dietary assessment tool

Participants' dietary intake was assessed using an online 168-items food frequency questionnaire (FFQ) whose reliability and validity has been approved previously (26). This questionnaire was used to collect data on dietary intake 1 year prior to the COVID-19 diagnosis in each patient. Participants initially reported their dietary intakes as daily, monthly, or annually. These data were then converted to grams per day (g/d) using household measures (27). The Nutritionist IV software was used to calculate energy and micro-and micronutrient intakes.

### COVID-19 severity assessment

The National Institutes of Health (NIH) COVID-19 Treatment Guideline (CTG) (28), updated on 19 October 2021, was used to determine the patients' COVID-19 severity. CTG classified the disease into five levels of severity; (1) Asymptomatic or presymptomatic infection: Individuals with a positive test for SARS-CoV-2 using either a nucleic acid amplification test (NAAT) or an antigen test but with no COVID-19 manifestations; (2) Mild illness: patients with any of the COVID-19 symptoms (e.g., loss of taste and smell, fever, headache, malaise, myalgia, nausea, vomiting, diarrhea, cough, sore throat) but no dyspnea or abnormal chest imaging; (3) Moderate illness: individuals with clinical evidence of lower respiratory tract involvement or chest imaging and an oxygen saturation (SpO_2_) of at least 94% on room air at sea level; (4) Severe illness: individuals with a SpO_2_ <94% on room air at sea level, a PaO_2_/FiO_2_ ratio <300 mmHg, a respiratory rate >30 breaths/min, or lung infiltrates >50%; and (5) Critical illness: individuals suffering from respiratory failure, septic shock, and/or multiple organ dysfunction. We assumed those who had mild and moderate diseases as non-severe.

### Assessment of COVID-19 symptoms

Participants were asked to complete a general questionnaire to obtain information about the presence of each common clinical manifestation of COVID-19 (i.e., fever, rigors, weakness, myalgia, dyspnea, cough, sore throat, nausea, and vomiting).

### Assessment of inflammatory markers

The initial measurement of C-reactive protein (CRP) at hospital admission was obtained from the medical records.

### Assessment of other variables

Data were collected on demographic characteristics, height, weight, physical activity, duration of convalescence, supplements intake, corticosteroids, or antiviral medications for each participant using the general questionnaire.

### Statistical analysis

The Kolmogorov–Smirnov test was used to assess the normal distribution of data (29). We divided participants into tertile groups based on their dietary intake of fruits, vegetables, and fiber. The energy-adjusted dietary intake of fruits, vegetables, and fiber using the residual method were calculated for this purpose (30). The means and SDs for continuous variables and the percentages for categorical variables were compared across tertiles of fruits, vegetables, and fiber intake using the one-way ANOVA and chi-square analysis, respectively. The inflammatory marker, i.e., CRP, were compared between tertiles of dietary intake of fruits, vegetables, and fiber using covariance analysis (ANCOVA) after being adjusted for age, gender, BMI, and physical activity. These variables were considered based on previous evidence (31, 32) and differences between tertiles of exposures of interest. Binary logistic regression was used in two models to explore the association between fruits, vegetables, and dietary fiber intake with odds of severe COVID-19 and also with the risk of each COVID-19 symptom. In the multivariable-adjusted model, age (continuous), energy intake (continuous), physical activity (sedentary/moderate/intense), and BMI (continuous) were adjusted. The Statistical Package for the Social Sciences (SPSS) was used for all data analyses (SPSS Inc., version 25). A *p*-value <0.05 was considered statistically significant.

## Results

A total of 250 patients were included in the final analysis, comprising 119 men and 131 women. The prevalence of mild disease was 5.6% (*n* = 14), the moderate disease was 48.4% (*n* = 121), and severe illness was 46% (*n* = 115) among subjects with a mean age of 44.2 years.

The general characteristics of study participants across tertiles of fruits, vegetables, and fiber intake are depicted in Table 1. Participants with higher intake of fruits, vegetables, and fiber were male with a lower BMI, and were less likely to be overweight or obese. In addition, they were less likely to use supplements or been prescribed corticosteroids or antiviral medications. After controlling for age, gender, BMI, and physical activity, participants in the top tertile of fruit intake had significantly lower CRP levels than those in the bottom tertile (10.51 ± 2.17 vs. 27.05 ± 2.25 mg/L). Similarly, participants in the highest tertile of vegetable and fiber intake had lower levels of CRP than those in the lowest tertile (12.92 ± 2.11 vs. 33.10 ± 2.10 mg/L, 10.95 ± 2.02 vs. 33.86 ± 2.12 mg/L, respectively). Furthermore, participants who consumed more fruits, vegetables, and fibers had a considerably shorter duration of hospitalization and convalescence.

**Table 1:** Demographic characteristics of the study participants across tertiles of dietary fruits, vegetables, and fiber.

	**Tertiles of fruits**					**Tertiles of vegetables**					**Tertiles of dietary fiber**			
	**T1**	**T2**	**T3**	**P^a^**		**T1**	**T2**	**T3**	**P^a^**		**T1**	**T2**	**T3**	**P^a^**
Participants (*n*)	83	84	83			83	84	83			83	84	83	
Age (years)	44.6 ± 12.0	45.3 ± 12.2	42.4 ± 12.2	0.26		43.8 ± 12.1	42.8 ± 13.1	45.8 ± 11.1	0.26		45.4 ± 12.4	43.0 ± 11.9	44.0 ± 12.2	0.43
Males (%)	42.2	41.7	59.0	0.04		44.6	42.9	55.4	0.21		43.4	45.2	54.2	0.33
BMI (kg/m^2^)	29.0 ± 3.8	26.0 ± 3.5	26.9 ± 3.7	<0.001		28.7 ± 3.5	26.8 ± 4.2	25.3 ± 2.6	<0.001		29.2 ± 3.6	25.8 ± 3.6	25.9 ± 3.0	<0.001
Physical activity				0.42					0.66					0.57
Sedentary (31)	15.7	14.3	7.2			12.0	15.5	9.6			16.9	11.9	8.4	
Moderate (202)	79.5	77.4	85.5			80.7	76.2	85.5			75.9	82.1	84.3	
Intense (17)	4.8	8.3	7.2			7.2	8.3	4.8			7.2	6.0	6.8	
Overweight or obese (%)	85.5	58.3	57.8	<0.001		86.7	67.9	47.0	<0.001		86.7	58.3	56.6	<0.001
Supplements intake (%)	98.8	96.4	89.2	0.01		95.2	96.4	92.8	0.56		98.8	95.2	90.4	0.04
Corticosteroids use (%)	98.8	92.9	84.3	0.003		94.0	91.7	90.4	0.68		98.8	89.3	88.0	0.02
Antiviral Drugs use (%)	98.8	92.9	84.3	0.003		94.0	91.7	90.4	0.68		98.8	89.3	88.0	0.02
CRP (mg/L)^b^	27.05 ± 2.25	21.37 ± 2.14	10.51 ± 2.17	<0.001		33.10 ± 2.10	13.01 ± 2.00	12.92 ± 2.11	<0.001		33.86 ± 2.12	14.21 ± 2.02	10.95 ± 2.02	<0.001
Duration of hospitalization (day)	7.6 ± 3.0	6.6 ± 2.8	5.4 ± 2.5	<0.001		7.4 ± 3.0	6.2 ± 3.1	6.0 ± 2.5	0.005		7.7 ± 2.9	6.2 ± 2.9	5.8 ± 2.7	<0.001
Convalescence duration (day)	11.3 ± 4.4	8.7 ± 3.3	8.3 ± 2.7	<0.001		10.5 ± 3.8	9.6 ± 4.3	8.2 ± 2.6	<0.001		11.3 ± 4.5	8.7 ± 3.2	8.3 ± 2.6	<0.001

All values are mean ± SD or percent.

*a*Obtained from ANOVA or chi-square test, where appropriate.

*b*Presented as mean ± SE, and values were adjusted for age, energy intake, BMI, and physical activity using ANCOVA.

**Figure 1:**
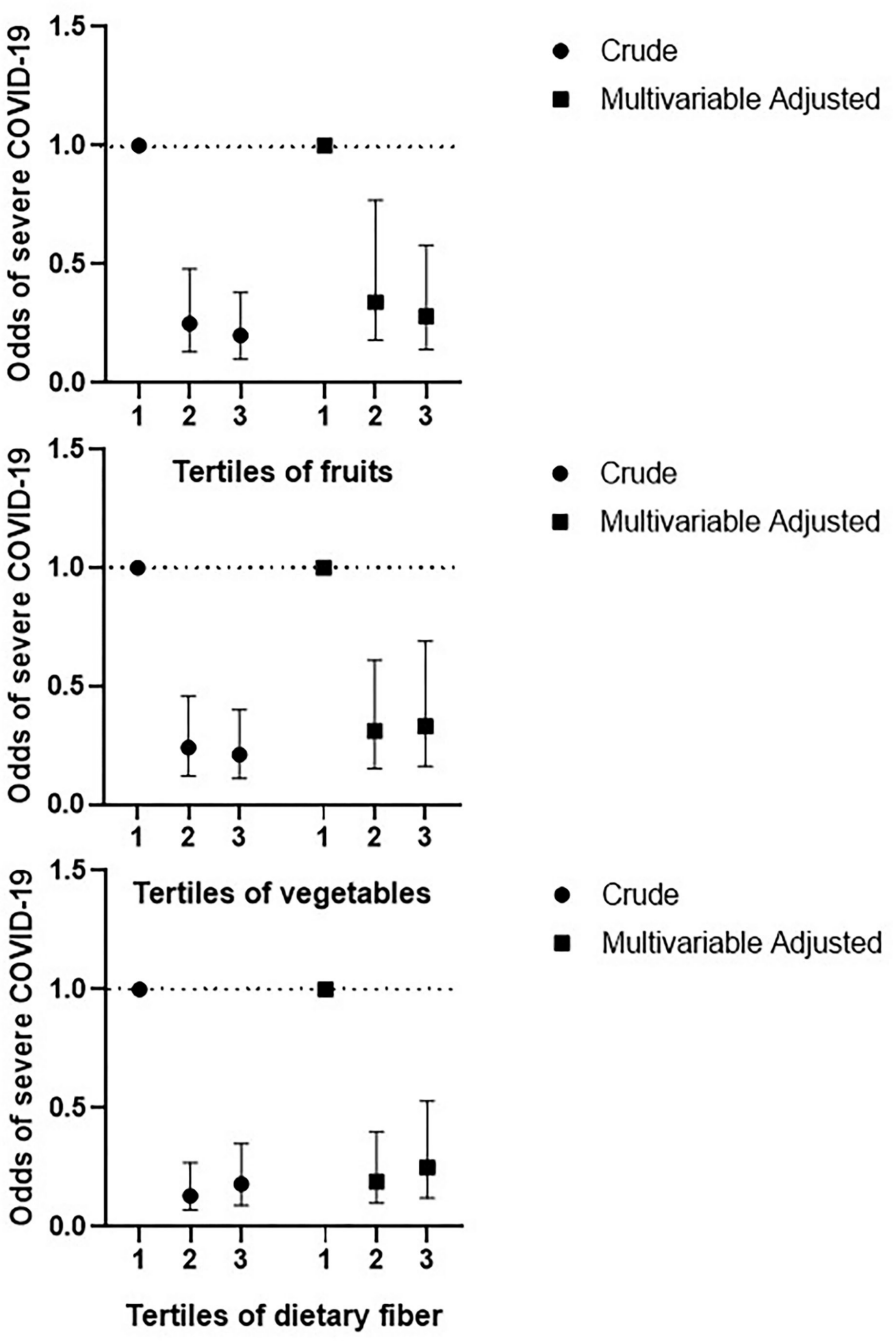
Crude and multivariable-adjusted odds ratios and 95% CIs of severe COVID-19 disease according to tertiles of dietary fruits, vegetables, and fiber. Multivariable adjusted for age, BMI, energy intake, and physical activity.

Crude and multivariable-adjusted odds ratios and 95% CIs for severe COVID-19 according to tertiles of dietary fruits, vegetables, and fiber intakes are illustrated in Figure 1. Participants in the highest tertile of fruits intake had a lower risk of severe COVID-19 than those in the lowest tertile either in the crude model (OR: 0.20; 95% CI: 0.10, 0.38, *P*-trend < 0.001) or after adjustment for potential confounders (OR: 0.28; 95% CI: 0.14, 0.58, *P*-trend < 0.001). In terms of vegetable intake, participants at the highest tertile had a lower risk of severe COVID-19, both in the crude model (OR: 0.21; 95% CI: 0.11, 0.40, *P*-trend < 0.001) and after adjusting for potential confounders (OR: 0.33; 95% CI: 0.16, 0.69, *P*-trend < 0.001). Similarly, participants at the highest tertile of fiber intake had a lower risk of severe COVID-19 in both crude model (OR: 0.18; 95% CI: 0.09, 0.35, *P*-trend < 0.001) and after adjusting for potential confounders (OR: 0.25; 95% CI: 0.12, 0.53, *P*-trend < 0.001).

Crude and multivariable-adjusted odds ratios and 95% CIs for COVID-19 symptoms according to tertiles of dietary fruits, vegetables, and fiber are reported in Table 2. After controlling for potential confounders, there was a significant inverse relationship between fruit consumption and the likelihood of experiencing respiratory COVID-19 symptoms such as dyspnea and cough, digestive symptoms such as nausea and vomiting, systemic-neurologic symptoms such as fever, chilling, and myalgia, and rhino pharyngeal symptoms such as sore throat. Participants in the highest tertile of vegetable consumption were significantly less likely to exhibit respiratory, digestive, systemic-neurologic, and rhino pharyngeal COVID-19 symptoms. Similar associations were found between dietary fiber consumption and COVID-19 symptoms.

**Table 2:** Crude and multivariable-adjusted odds ratios and 95% CIs for symptoms of COVID-19 according to tertiles of dietary fruits, vegetables, and fiber.

	**Tertiles of fruits**					**Tertiles of vegetables**					**Tertiles of dietary fiber**			
	**T1**	**T2**	**T3**	$P_{\text{trend}}^{*}$		**T1**	**T2**	**T3**	$P_{\text{trend}}^{*}$		**T1**	**T2**	**T3**	*P* ${}_{\text{trend}}^{*}$
**Dyspnea**														
Crude	1.00	0.26 (0.13, 0.53)	0.21 (0.10, 0.43)	<0.001		1.00	0.40 (0.20, 0.79)	0.25 (0.13, 0.50)	<0.001		1.00	0.11 (0.05, 0.25)	0.18 (0.08, 0.39)	<0.001
Model 1	1.00	0.34 (0.15, 0.74)	0.28 (0.13, 0.60)	0.002		1.00	0.48 (0.23, 1.00)	0.37 (0.17, 0.77)	0.01		1.00	0.14 (0.06, 0.33)	0.23 (0.10, 0.54)	0.004
**Cough**														
Crude	1.00	0.11 (0.05, 0.25)	0.12 (0.06, 0.26)	<0.001		1.00	0.27 (0.14, 0.53)	0.20 (0.10, 0.39)	<0.001		1.00	0.07 (0.03, 0.17)	0.11 (0.05, 0.24)	<0.001
Model 1	1.00	0.16 (0.07, 0.36)	0.17 (0.07, 0.38)	<0.001		1.00	0.34 (0.16, 0.70)	0.35 (0.17, 0.75)	0.009		1.00	0.10 (0.04, 0.24)	0.15 (0.06, 0.35)	<0.001
**Fever**														
Crude	1.00	0.13 (0.05, 0.35)	0.12 (0.05, 0.37)	<0.001		1.00	0.09 (0.03, 0.27)	0.10 (0.03, 0.31)	<0.001		1.00	0.09 (0.03, 0.26)	0.11 (0.04, 0.34)	<0.001
Model 1	1.00	0.15 (0.05, 0.50)	0.15 (0.05, 0.43)	0.001		1.00	0.10 (0.03, 0.31)	0.13 (0.04, 0.42)	<0.001		1.00	0.10 (0.03, 0.32)	0.13 (0.04, 0.41)	0.002
**Chilling**														
Crude	1.00	0.07 (0.02, 0.26)	0.07 (0.02, 0.24)	<0.001		1.00	0.07 (0.02, 0.23)	0.08 (0.02, 0.27)	<0.001		1.00	0.04 (0.01, 0.18)	0.05 (0.01, 0.22)	<0.001
Model 1	1.00	0.09 (0.02, 0.34)	0.09 (0.02, 0.31)	<0.001		1.00	0.08 (0.02, 0.27)	0.10 (0.03, 0.38)	0.002		1.00	0.05 (0.02, 0.23)	0.06 (0.02, 0.28)	<0.001
**Weakness**														
Crude	1.00	0.65 (0.34, 1.22)	0.34 (0.17, 0.69)	0.003		1.00	0.40 (0.20, 0.77)	0.25 (0.12, 0.50)	<0.001		1.00	0.34 (0.17, 0.65)	0.26 (0.13, 0.51)	<0.001
Model 1	1.00	1.00 (0.48, 2.10)	0.54 (0.24, 1.16)	0.11		1.00	0.44 (0.22, 0.89)	0.31 (0.14, 0.69)	0.003		1.00	0.46 (0.22, 0.94)	0.35 (0.17, 0.74)	0.006
**Myalgia**														
Crude	1.00	0.66 (0.36, 1.22)	0.29 (0.14, 0.55)	<0.001		1.00	0.49 (0.27, 0.92)	0.35 (0.18, 0.66)	0.001		1.00	0.55 (0.30, 1.01)	0.31 (0.16, 0.59)	<0.001
Model 1	1.00	0.78 (0.39, 1.53)	0.41 (0.20, 0.83)	0.01		1.00	0.60 (0.31, 1.16)	0.44 (0.21, 0.90)	0.02		1.00	0.78 (0.39, 1.53)	0.41 (0.20, 0.83)	0.01
**Nausea and vomiting**														
Crude	1.00	0.84 (0.39, 1.82)	0.05 (0.01, 0.36)	<0.001		1.00	0.16 (0.06, 0.43)	0.12 (0.04, 0.38)	<0.001		1.00	0.16 (0.06, 0.43)	0.12 (0.04, 0.38)	<0.001
Model 1	1.00	1.21 (0.48,0.05)	0.06 (0.01, 0.52)	0.005		1.00	0.15 (0.05, 0.44)	0.15 (0.05, 0.51)	<0.001		1.00	0.18 (0.06, 0.54)	0.13 (0.04, 0.45)	<0.001
**Sore throat**														
Crude	1.00	0.56 (0.30, 1.05)	0.15 (0.07, 0.34)	<0.001		1.00	0.51 (0.27, 0.95)	0.15 (0.07, 0.32)	<0.001		1.00	0.25 (0.13, 0.49)	0.12 (0.06, 0.26)	<0.001
Model 1	1.00	0.32 (0.16, 0.66)	0.16 (0.07, 0.36)	<0.001		1.00	0.59 (0.30, 1.14)	0.19 (0.08, 0.45)	<0.001		1.00	0.32 (0.16, 0.66)	0.16 (0.07, 0.35)	<0.001

Adjusted for age, BMI, energy intake, and physical activity.

*Obtained from Binary logistic regression.

## Discussion

Our study investigated the association between dietary intake of fruits, vegetables, and fiber and odds of severe COVID-19 and also COVID-19 symptoms among 250 hospitalized Iranian adults. According to our findings, the severity of COVID-19 and related symptoms was lower in those with a greater consumption of fruits, vegetables, and fibers. In addition, the requirement for corticosteroids and antiviral medications, the days of hospitalization and convalescence, and level of inflammation marker, i.e., CRP, were notably reduced in participants who consumed more fruits, vegetables, and fibers.

Health benefits of a rich diet in vegetables, fruits and fiber have long been investigated in previous studies. The Mediterranean diet, which contains high quantities of the aforementioned dietary elements, has been shown to have anti-inflammatory and antioxidant potential, as well as to ensure optimal immune activity, and its use has been recommended as a preventive strategy in the development of cardiometabolic diseases, namely, obesity, cardiovascular disease, type 2 diabetes mellitus, malignancies, asthma, allergies, and respiratory tract infections (28, 33). Our findings support this hypothesis, as subjects who followed a dietary pattern similar to the Mediterranean diet had fewer symptoms and less severe forms of COVID-19, and also lower levels of inflammatory markers. On the other hand, it has been demonstrated that the Western diet, which is high in fats and carbohydrates and low in fibers and antioxidants, is pro-inflammatory, stimulates innate immunity and the production of reactive oxygen species, and diminishes the efficacy of the adaptive immune response (34). Micronutrients and bioactive natural compounds, such as vitamins A, C, D, and/or E, minerals (iron, selenium, and zinc), probiotics, fibers, polyphenols, and omega-3 polyunsaturated fatty acids, have been shown to enhance the immune system's antiviral response and prevent the development of respiratory tract infections (35–37).

There is no association between the risk of upper respiratory tract infections and other dietary patterns, such as the Nordic dietary pattern issued by a joint committee of experts from the Nordic countries (38). Although the potential benefits of a Mediterranean-style diet in protecting against COVID-19 have been hypothesized, there is a lack of clinical evidence to support these theories. This is primarily due to the diet's antioxidant and anti-inflammatory effects, which can counteract the pro-oxidant and pro-inflammatory status triggered by the SARS-CoV-2 infection (3). The results of our cross-sectional study have, to the best of our knowledge, revealed for the first time an association between a higher intake of vegetables, fruits, and fiber and milder symptoms and less severe forms of COVID-19. In addition, patients who consumed more of these foods had shorter hospital stays, required fewer days to recover, and had lower CRP levels as inflammatory markers. Similarly, Merino et al. (4) investigated the dietary pattern of nearly 32,000 COVID-19 subjects and delineated that individuals who followed a plant-based nutritional approach were less likely to get infected with SARS-CoV-2 or may have a milder form of this respiratory infection (4). In addition, Salazar-Robles (39) concluded that the consumption of vegetables and grains was associated with COVID-19 symptoms that were less severe. According to an evaluation of 236 outpatients from Mexico, 44% tested positive for SARS-CoV-2 (39).

Elevated oxidative stress and inflammation levels in COVID-19 can result in a dysfunctional immune system response (40). Phytochemicals and antioxidants from fruits and vegetables can eliminate pro-oxidant and pro-inflammatory molecules associated with viruses. In addition, it can affect the interaction between oxidative stress and the NF-kB and/or Nrf-2 pathways (41). The mechanisms mentioned earlier could explain who consumed a lot of vegetables, fruits, and fibers in our study had lower levels of inflammation markers and were infected with a milder form of SARS-CoV-2. Furthermore, nutrients in fruits and vegetables may interact favorably with the gut microbiota to counteract excessive inflammation (42). The intestinal microbiome converts dietary fibers into short-chain fatty acids, which has lower inflammatory activity (41). Therefore, it is hypothesized that a diet rich in fruits and vegetables may protect against the hyperinflammation and cytokine storm observed in severe COVID-19 forms (43, 44). As a result, the COVID-19 lockdowns and other events associated with the SARS-CoV-2 pandemic may have influenced the food choices of our participants, as our study was based on data derived from the hospital records of patients with COVID-19 hospitalized between June and September 2021.

Our study has several strengths and limitations. To the best of our knowledge, this is the first cross-sectional study reporting the effect of diet on COVID-19 symptomatology, severity, and laboratory parameters. The findings of our study can be used to issue dietary recommendations by experienced dietitian, researchers, physicians, and other clinical nutrition experts during the COVID-19 pandemic. Another strength is that we recruited people up to the age of 65, representing the great majority of hospitalized patients with SARS-CoV-2 infection. The elderly may be challenging to recollect their dietary consumption precisely; hence, their inclusion may affect our judgments. However, several limitations must be considered. First, due to the study's cross-sectional nature, causality cannot be determined. Future research should evaluate the association between vegetables, fruits, and fiber consumption and COVID-19 symptomatology and severity; ideally in prospective cohort studies with larger study samples. Second, we used a validated and reliable questionnaire to assess the dietary intake; however, misclassification could not be completely excluded. Third, despite accounting for a number of potential confounding variables, residual confounding cannot be excluded. Fourth, the study sample was relatively small considering the number of patients diagnosed with COVID-19 and was only limited to one city in Iran, thus affecting the generalization of the results for entire Iran or other countries. Fifth, the population of the study was restricted to healthy adults, so the findings cannot be generalized to other groups. For a better understanding of this relationship, it is suggested that additional research be conducted on populations with underlying diseases such as diabetes and cardiovascular disease. Finally, dietary alterations might have been induced by the COVID-19 pandemic, and the fear of contracting this infectious disease, and, thus, the investigated patients might only temporarily adhere to healthy eating habits.

## Conclusion

We found that patients with a higher intake of fruits, vegetables, and fibers had a decreased likelihood of severe form of COVID-19 and related symptoms. In addition, the need for corticosteroids and antiviral medications, the length of hospitalization, and convalescence, and also the levels of the inflammatory marker, i.e., CRP, were significantly lower in patients who consumed more fruits vegetables, and fibers. Due to the limitations highlighted earlier, our findings should be regarded with caution, and it is recommended that this association be evaluated in large prospective studies and randomized trials.

## Data availability statement

The raw data supporting the conclusions of this article will be made available by the authors, without undue reservation.

## Ethics statement

The Ethics Committee of Kashan University of Medical Sciences reviewed and authorized studies involving human participants. The patients/participants provided their written informed consent to participate in this study.

## Author contributions

AM and MT conceived, designed, and supervised the study. AE and CA contributed to data collection. KT, NZ, SM, and M-AG performed statistical analyses, data interpretation, and drafting of the manuscript. PMob, PMok, and HR contributed to the manuscript drafting and editing. All authors contributed to the article and approved the submitted version.

## Conflict of interest

The authors declare that the research was conducted in the absence of any commercial or financial relationships that could be construed as a potential conflict of interest.

## Publisher's note

All claims expressed in this article are solely those of the authors and do not necessarily represent those of their affiliated organizations, or those of the publisher, the editors and the reviewers. Any product that may be evaluated in this article, or claim that may be made by its manufacturer, is not guaranteed or endorsed by the publisher.
